# Cystatin C estimated glomerular filtration rate to assess renal function in early stages of autosomal dominant polycystic kidney disease

**DOI:** 10.1371/journal.pone.0174583

**Published:** 2017-03-27

**Authors:** Laia Sans, Aleksandar Radosevic, Claudia Quintian, Rosario Montañés, Silvia Gràcia, Carles Vilaplana, Sergi Mojal, José A. Ballarin, Patricia Fernández-Llama, Roser Torra, Julio Pascual

**Affiliations:** 1 Department of Nephrology, Hospital del Mar, Barcelona, Spain; 2 Institut Mar for Medical Research, Barcelona, Spain; 3 Red Temática de Investigación Cooperativa (RedinRen), Instituto Carlos III, Madrid, Spain; 4 Department of Radiology, Hospital del Mar, Barcelona, Spain; 5 Department of Radiology, Fundació Puigvert, Barcelona, Spain; 6 Laboratory Department, Fundació Puigvert, Barcelona, Spain; 7 Laboratory Department, Laboratori de Referència de Catalunya, Barcelona, Spain; 8 Department of Nephrology, Fundació Puigvert, Barcelona, Spain; 9 Universitat Autònoma de Barcelona. Instituto de Investigación Biomédica Sant Pau (IIB Sant Pau), Barcelona, Spain; Hospital Universitario de la Princesa, SPAIN

## Abstract

**Background/Aims:**

Height-adjusted total kidney volume (htTKV) is the best marker of disease progression in early autosomal dominant polycystic kidney disease (ADPKD) when renal function still remains normal. The usefulness of cystatin-C as a biomarker to assess renal function according to renal volume has not been studied in ADPKD patients.

**Methods:**

Observational and cross-sectional study of 62 ADPKD patients. htTKV, creatinine and cystatin-C estimated glomerular filtration rate (eGFR) were determined. Correlations between htTKV and eGFR were studied. A control group was used to determine the association between renal function differences and htTKV.

**Results:**

htTKV significantly correlated with cystatin-C-eGFR (r = -0.384, p = 0.002) but not with creatinine-eGFR (r = -0.225, p = 0.078). With htTKV stratified into tertiles, a significant difference of cystatin-C-eGFR but not in creatinine-eGFR was detected in the third tertile when compared with the first tertile group (110.0±22.2 vs 121.3±7.2; p = 0.023 and 101.8±17.2 vs 106.9±15.1; p = 0.327 respectively). When cystatin-C-eGFR of the controls was used as the reference, htTKV above 605 ml/m identified with a 75% sensitivity and 84.9% specificity those patients with a significant worse kidney function. However, this cut-off value could not be identified using creatinine-eGFR.

**Conclusions:**

Cystatin-C-eGFR but not creatinine-eGFR correlated with htTKV in ADPKD patients in early stages of the disease. Differences in cystatin-C-eGFR but not in creatinine-eGFR have been identified through htTKV tertiles. A htTKV above 605 ml/m is associated with a worse renal function only if cystatin-C-eGFR is used. Cystatin-C-eGFR should be studied in prospective studies of early stages of ADPKD to determine its usefulness as an early marker of disease progression.

## Introduction

Autosomal dominant polycystic kidney disease (ADPKD) is the most common renal inherited disorder. Mutations in *PKD1* and *PKD* 2 genes (encoding for polycystin 1 (PC1) and polycystin 2 (PC2)) lead to the formation and progressive expansion of kidney cysts which ultimately cause kidney enlargement and distortion of renal architecture, thus leading to kidney failure[1].

Glomerular filtration rate (GFR) is considered the gold standard for quantifying the rate of progression in the vast majority of renal disorders. Since it is rarely measured in clinical practice, estimated GFR (eGFR) equations are usually applied to monitor renal function decline. Notably, renal function seems to remain stable during the first decades of life in ADPKD patients, even though the normal renal parenchyma is gradually replace by cysts[2]. There is a weak relationship between renal function and renal volume at the beginning of the disease but this relationship becomes stronger as the disease progresses[3]. Because creatinine and eGFR equations based on creatinine are not suitable for monitoring the progression of the disease in early stages[3], renal volume has been identified as the best method for this purpose and has become the primary outcome in all randomized clinical trials in early stages of ADPKD. The gold standard for the assessment of renal volume is nuclear magnetic resonance, which is not easily available for all the clinicians in their daily practice. The assessment of renal volume using ultrasound is feasible in early stages of the disease but requires some expertise and even though it is a good method for risk stratification, it is less useful for the periodic monitoring of the disease[4,5].

Contemporary research suggests that cystatin C may be an improved alternative to creatinine for inclusion in eGFR equations because it has fewer non-GFR determinants and is especially helpful when eGFR is still in the normal range[6]. To our knowledge, the relationship between renal volume and eGFR using equations with cystatin C in early stages of ADPKD has not been studied before. Therefore, in this study we want to analyze if Cystatin C could represent an easier method to monitor disease progression in early stages of the disease.

## Methods

This study was conducted after the approval of the Ethics Committee of Hospital del Mar and Fundació Puigvert and was performed according to the principles expressed in the Declaration of Helsinki. Informed consent was signed by all participants.

It is a subanalysis of an observational and cross-sectional study whose main objective was to determine whether the increase in renal volume was associated with a worsening of the cardiovascular profile in normotensive ADPKD patients with normal renal function compared with a control group. For the assessment of renal function CKD-EPI eGFR was calculated based on creatinine and cystatin C.

The relationship between renal volume and CKDEPI eGFR with creatinine and cystatin C equations in early stages of ADPKD was evaluated.

### Study population

Patients were recruited from the outpatient clinic from two University tertiary-care hospitals, Hospital del Mar and Fundació Puigvert, both in Barcelona, Spain. ADPKD diagnostic was based in Pei’s ultrasonographic criteria if patients had a positive family history[7]; otherwise patients with bilaterally enlarged kidneys with innumerable cysts in the absence of other findings to suggest a different cystic disease were considered to have ADPKD[8].

Patients were included in the study if they were not under antihypertensive treatment and office blood pressure (oBP) and home blood pressure (HBPM) were within the normal range (≤140/90 mmHg for oBP and ≤135/85 mmHg for HBPM) until the last medical visit before the inclusion and had normal renal function (CKD-EPI eGFR> 60 ml/min/1.73m^2^).

A control group of healthy volunteers and individuals evaluated as living kidney donors matched by age and renal function with the ADPKD group were also included.

### Study protocol

Ultrasound renal volume was measured with the ellipsoid formula[9] using a General Electric LOGIC^®^ ultrasound (General Electric Medical Health, Waukesha, WI) with a 3,5mHz probe by an experienced radiologist in each center both following the same protocol. To summarize, longitudinal diameter was recorded and anterior-posterior and transverse diameter were measured with a cross sectional image of the kidney at the hilum level[5]. All ultrasounds were performed by expert radiologists in each center used to follow up ADPKD patients with ultrasound. Inter -explorer variability was previously assessed. The two radiologists showed a variability intra and inter-observer of less than 5% in TKV measurement. Total renal volume was adjusted to height (htTKV).

Morning blood sample was drawn and serum creatinine was measured by a Jaffe reaction (Modular P; Roche Diagnostics®, Mannheim, Germany) traceable to isotope dilution mass spectrometry. Serum Cystatin C was measured (Roche Diagnostics®, Manheim, Germany) with an immunoturbidimetric assay. eGFR was calculated using CKD-EPI equations for creatinine and cystatin C as follows [10,11]:

*serum creatinine standardized* *≤0.7 mg/dl for women = 144 × (creatinine/0.7^)-0.329^ × (0.993)^age^*
*serum creatinine standardized* *>0.7 mg/dl for women = 144 × (creatinine/0.7)^-1.209^ × (0.993)^age^*
*serum creatinine standardized* *≤0.9 mg/dl for men = 141 × (creatinine/0.9)^-0.411^ × (0.993)^age^*
*serum creatinine standardized* *>0.9 mg/dl for men = 141 × (creatinine/0.9)^-1.209^ × (0.993)^age^*
*serum cystatin C standardized* *≤ 0.8 mg/L = 133 x (Cystatin C/ 0.8)^-0.499^ x 0.996 ^(age)^ x 0.932 (if woman)*
*serum cystatin C standardized* *> 0.8 mg/L = 133 x (Cystatin C/ 0.8)^-1.328^ x 0.996 ^(age)^ x 0.932 (if woman).*

### Statistical analysis

Mean±SD and median ([interquartilic range [IQR]) were used to express the results for parametric and non-parametric variables respectively, and categorical variables results are shown as percentage. Log transformation was applied to those variables not normally distributed. T student and ANOVA were used to compare means for normally distributed variables while for non parametric, U-Mann-Whitney or Kruskall-Wallis were applied for comparisons between two or more groups. Differences in categorical variables were studied with Chi^2^ test. Associations between variables were determined through Pearson correlation or Spearman coefficient. With receiver operating characteristic curve (ROC) the renal volume cut off point with better sensitivity and specificity to predict loss of renal function was determined. SPSS 21 (SPSS®, Chicago, IL, USA) was used to perform the statistical analysis. *P* values less than 0.05 were considered statistically significant.

## Results

### Study population characteristics

Sixty-two ADPKD patients and 28 healthy controls were studied. Baseline characteristics are shown in Table 1. There were no differences in terms of age and gender between patients and controls. Patients showed normal renal function using both creatinine and cystatin eGFR equations and no differences with the control group were found. The ADPKD group showed larger htTKV when compared with control group.

**Table 1:** Basal characteristics of adult dominant polycystic kidney disease patients and a control group.

	ADPKD (n = 62)	Control (n = 28)	*p*
Age (years)	33.9 ± 8.5	32.3 ± 7.5	0.381
Gender (males) (%)	40.3	42.9	0.823
Body mass index (kg/m^2^)	23.4 ± 3.5	23.5 ± 3.9	0.876
Serum creatinine (mg/dL) *(mmol/L)*	0.81 ± 0.17 *(71*,*6 ± 15*,*0)*	0.79 ± 0.14 *(69*,*8 ± 12*,*37)*	0.624
Creatinine eGFR * (ml/min/1,73m^2^)	105.1 ± 14.7	108.3 ± 10.2	0.305
Serum cystatin C (mg/L)	0.72 ± 0.15	0.73 ± 0.12	0.752
Cystatin C eGFR * (ml/min/1,73m^2^)	116.3 ± 16.4	116.7 ± 13.7	0.907
htTKV (ml/m)	402 [303–567]	158 [134–195]	**<0.001**
Renal diameter (cm)	13.8 ± 2.0	10.2 ± 0.6	**<0.001**

* Using CKD-EPI equations. htTKV: height adjusted total kidney volume. Results are expressed by mean ± standard deviation and median [interquartile range]

### Estimation of GFR with creatinine and cystatin C derived equations and correlation with renal volume

**Fig 1:**
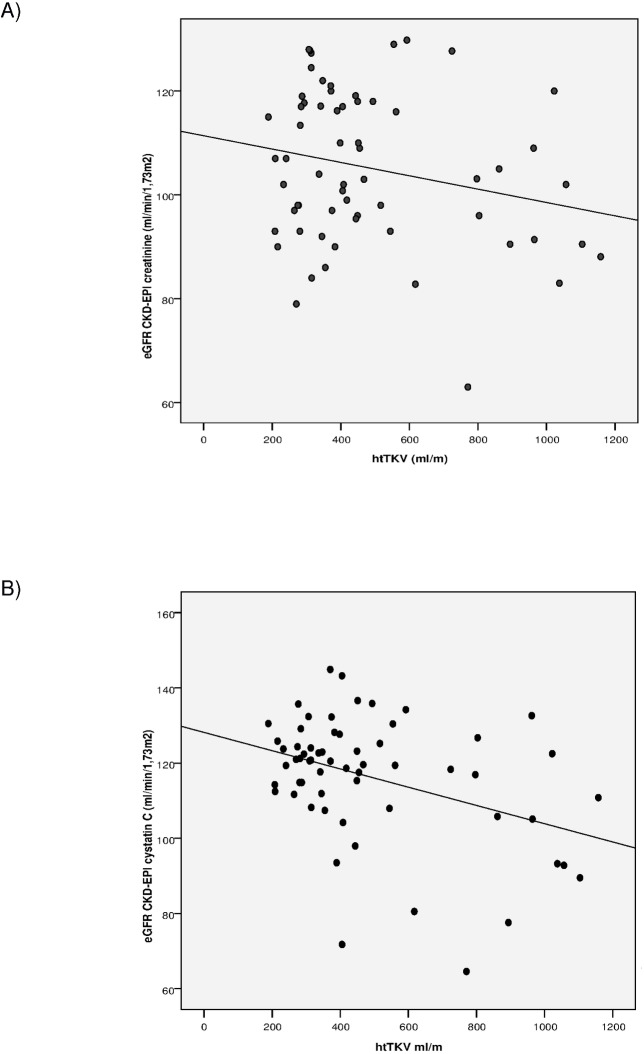
Correlation between htTKV and eGFR in ADPKD group. A) With the creatinine-derived equation. B) With the Cystatin C—derived—equation.

In the ADPKD group creatinine-eGFR and cystatin-C-eGFR showed a significant correlation (r = 0.557, p<0.001). The correlation between renal diameter and renal volume was good (r = 0.840, p<0.001). Correlations between eGFR and htTKV were studied in the ADPKD group. When creatinine was used, htTKV did not show a significant correlation with eGFR (r = -0.225, p = 0.078) while the correlation between htTKV and eGFR using cystatin C was statistically significant (r = -0.384, p = 0.002) (Fig 1).

Taking into account that htTKV has been demonstrated to be a good marker for risk stratification, we classified patients into htTKV tertiles and we assessed renal function according to each tertile group. The first tertile group included patients with a htTKV below 336.5 ml/m htTKV (n = 20), second tertile group between 336.5 and 467.5 ml/m (n = 21) and the third tertile above 467.5 ml/m (n = 21). Renal function according to tertiles is shown in Table 2. Serum creatinine or creatinine-eGFR levels were similar in first and second tertile groups. The second tertile group showed a slight increase of cystatin C levels and lower cystatin-C-eGFR, but the differences did not reach statistical significance. Patients in the third tertile group did not show changes neither in creatinine levels nor in creatinine-eGFR when compared with the first tertile group. Nevertheless when renal function was assessed using cystatin C and cystatin-C-eGFR, patients in the third tertile group showed a significantly worse renal function than patients in the first tertile group (p = 0.023), even though renal function remained normal. In other words, when renal function was assessed with cystatin C instead of creatinine, a statistical significant difference of renal function was determined through renal volume tertiles.

**Table 2:** Renal function of ADPKD patients according to htTKV tertiles.

	1^st^ tertile htTKV	2^nd^ tertile htTKV	3^rd^ tertile htTKV	*p* 1^st^ vs 2^nd^	*p* 2^n^ vs 3^rd^	*p* 1^st^vs 3r^d^
**Serum Creatinine** (mg/dL) *(mmol/l)*	0.78±0.15 *(68*,*9±13*,*3)*	0.79±0.15 *(69*,*8±13*,*3)*	0.87±0.19 *(76*,*9±16*,*8)*	0.875	0.156	0.125
**Creatinine-eGFR** (ml/min/1,73m^2^)	106.9±15.1	106.7±11.4	101.8±17.2	0.973	0.285	0.327
**Serum Cystatin-C** (mg/L)	0.67±0.07	0.70±0.15	0.79±0.18	0.497	0.094	**0.011**
**Cystatin-C-eGFR** (ml/min/1,73m^2^)	121.3±7.2	117.9±17.3	110.0±20.2	0.414	0.187	**0.023**

htTKV: Height-adjusted total kidney volume. Results are expressed by mean ± standard deviation

### Renal volume and kidney function

**Fig 2:**
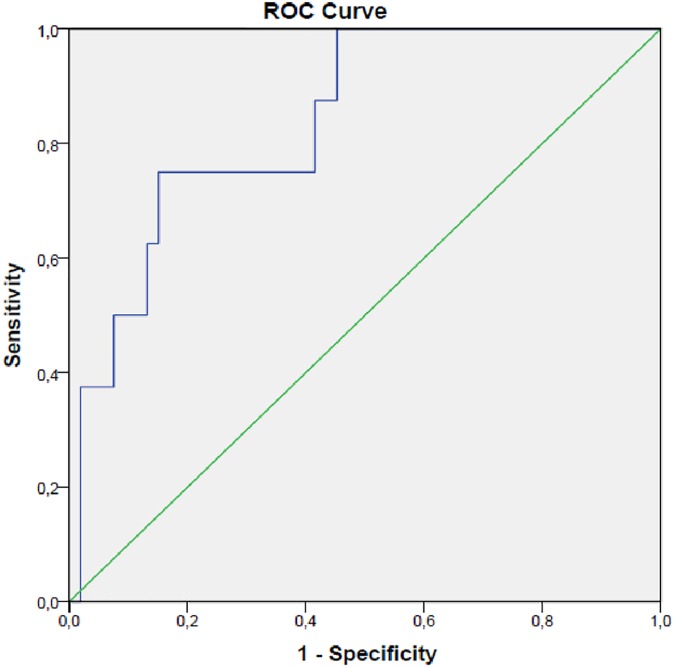
ROC curve for a htTKV of 605 ml/m associated with a loss of renal function using eGFR cystatin C.

htTKV is the best method for monitoring disease progression in the early stages of the disease. We studied if there was a renal volume cut off value that could identify those patients with a significant worse renal function even though in 1–2 CKD stage. A creatinine-eGFR of 89.4 ml/min/1.73m^2^ and a cystatin-C-eGFR of 95.6 ml/min/1.73m^2^ corresponded with the 5^th^ percentile renal function of the healthy control group studied and were used as the renal function reference value. A receiver operating characteristic curve was performed using both cut off values. When cystatin-C-eGFR was used, a htTKV of 605 ml/m was identified as the limit beyond which a worse renal function was detected in ADPKD patients. A htTKV of 605 ml/m with an area under the curve of 0.840 (95% CI 0.708–0.971; p = 0.002) was associated with a sensitivity of 75% and a specificity of 84.9% with a worse renal function when the 5^th^ percentile of renal function of the control group was used as a reference (Fig 2). When the same analysis was done using creatinine-eGFR a cut off htTKV could not be identified.

## Discussion

Total renal volume has become the most important tool for monitoring the progression of ADPKD in early stages of the disease[12]. During the first decades of the disease renal function remains stable even though kidneys experience a progressive enlargement due to the appearance and growing of renal cysts[2]. Consequently, the correlation between renal volume and renal function has been described as being weak. As disease progresses, the correlation between renal volume and renal function becomes stronger[3]. Renal volume has become the main outcome to test the efficacy of novel treatments in early stages of the disease in clinical trials[13–15]. As some strategies have shown beneficial results delaying renal enlargement [14], they have mostly failed to demonstrate reduction in loss of kidney function when different methods and equations derived of creatinine (creatinine plasma concentrations, Cockroft–Gault, MDRD, CKD-EPI, 1/creatinine) have been used[13,15]. These discordant results might be explained by this weak correlation between renal volume and renal function in early stages, and by an inability of creatinine derived methods to detect early changes in renal function in ADPKD.

Creatinine is the endogen marker used to assess renal function but the sensitivity of creatinine to detect early loss of renal function is not sufficient as its serum concentration remains stable until the GF does not get under 50% of its reference target. Moreover, the estimation of GFR taking into account serum creatinine levels might overestimate the real GFR because of the tubular secretion of creatinine[6].

In ADPKD patients at high measured GFR (>105 ml/min/1.73m^2^), tubular secretion of creatinine has been shown to be significantly greater than in healthy controls with the same renal function, thus contributing to a lower plasma creatinine level at those early stages of the disease[16]. Nowadays CKD-EPI equation has been shown to be the most precise equation to estimate GFR especially for those with eGFR values >60ml/min/1.73m2[17]. In adult ADPKD patients with normal renal function it is the equation with better accuracy taking measured GFR as the gold standard[18].

Cystatin C is a 13kDA molecule which filters from glomerulus and is catabolized (but not secreted) from proximal renal tube. Reductions in GFR are linked to increases in serum cystatin C. Serum levels of cystatin C, contrary to serum creatinine levels, are not affected by age, gender, race o muscle mass, thus makes it a better biomarker to estimate renal function loss in critically ill patients or in the elderly[6]. Moreover, serum cystatin C has been reported to increase as the GFR falls below about 80/ml/min/1.73m^2^, and therefore is particularly useful when trying to detect mild impairment of renal function[11].

The use of cystatin C for the evaluation of renal function has been very limited in ADPKD patients. It has been reported that when renal function is still normal, eGFR equations which include the use of both creatinine and cystatin C as biomarkers of renal function show better accuracy with measured GFR than equations of eGFR that only use creatinine[18]. The relationship between renal volume and cystatin C has never been studied before, but an ultrasound score of the severity of the disease (using the number of kidney cysts and renal enlargement) has not shown any correlation with serum cystatin C concentrations[19]. In our study, using CKD-EPI derived equations, we have found a significant correlation between cystatin-C-eGFR and renal volume, but no correlation was found using creatinine-eGFR. Moreover, when we classified patients into renal volume tertiles, we found significant differences in renal function when measured with cystatin C through renal volume tertiles but this difference of renal function was not significant when creatinine was used. These findings may point towards cystatin C being an earlier marker to detect kidney loss in early stages of ADPKD. Finally, when we used the renal function of the healthy population studied (based on cystatin C-eGFR) as the reference normal renal function, we identified a htTKV above 605 ml/m as the cut off renal volume associated with a worse renal function. However, this cut off value could not be found when the renal function was studied using creatinine eGFR. Interestingly, data from the cohort of the CRISP study pointed towards a htTKV of 600ml/m as the renal volume which predicted the progression towards renal insufficiency in 8 years follow-up[3].

Our study has some limitations. The number of patients is small, and our results should be confirmed in larger populations. The cross-sectional nature of our study, precluded assessing the usefulness of the cystatin C-eGFR equation in a longitudinal follow-up, and it should be noticed that we lack measured GFR as the gold standard to assess renal function. The use of ultrasound to determine renal volume may raise some criticism. The gold standard for renal volume assessment, especially for monitoring changes in renal volume, is nuclear magnetic resonance[20]. However, in daily clinical practice nuclear magnetic resonance is rarely used. Renal ultrasound is the mostly used imaging technique, and efforts have been made in evaluating the usefulness of renal enlargement assessed by ultrasound. Renal volume and length measured by ultrasonography, when kidneys are only slightly enlarged, have been demonstrated to be a good stratification method for at risk patients of progression, and correlation between renal volume and diameter both evaluated by ultrasound is extremely good when renal diameter is less than 17 cm[5]. Moreover, ultrasound renal volume shows a good correlation with magnetic resonance renal volume in early stages of the disease[21]. In this study, we included patients at very early stages of the disease. We have found a good correlation between renal diameter and renal volume and the mean renal diameter of our population was clearly below 17 cm. Moreover, we have used kidney volume to stratify the risk and not to evaluate volume progression. Therefore, we consider that evaluation of renal volume by ultrasound in this study is sufficient for our purpose. Finally, the fact that cystatin C has also been shown to be a biomarker of cardiovascular risk [22]could represent an interfering factor in this renal volume–eGFR cystatin C relationship. Even though, the absence of hypertension among the group of patients studied might reduce the risk of bias.

To our knowledge, this is the first study to assess the correlation between renal function using cystatin C and renal volume in ADPKD. The reported results might point towards cystatin C being a good biomarker to evaluate renal function and loss of renal function in early stages of the disease. The use of cystatin C as a marker of disease progression in early stages might represent an easier marker to be used than renal volume, which represents the best method for disease monitoring when renal function remains normal. Even though, the results of this study should be confirmed in prospective studies.

## Conclusions

The results of this study point towards a better correlation of htTKV with cystatin-C-eGFR than with creatinine-eGFR. When patients are classified according to their htTKV, accepted as the best marker of disease progression at early stages of the disease, differences in cystatin-C-eGFR but not in creatinine-eGFR are noticed, which might point towards cystatin-C as a biomarker to monitor disease progression in early stages of the disease.
